# New Strategy for the Degradation of High-Concentration Sodium Alginate with Recombinant Enzyme 102C300C-Vgb and the Beneficial Effects of Its Degradation Products on the Gut Health of *Stichopus japonicus*

**DOI:** 10.3390/md23090339

**Published:** 2025-08-25

**Authors:** Ziqiang Gu, Feiyu Niu, Peng Yang, Wenling Gong, Hina Mukhtar, Siyu Li, Yanwen Zheng, Yiling Zhong, Hanyi Cui, Jichao Li, Haijin Mou, Dongyu Li

**Affiliations:** College of Food Science and Engineering, Ocean University of China, No.1299 Sansha Road, Qingdao 266003, China; ziqianggu1568@163.com (Z.G.); 15686290590@163.com (F.N.); 15171165896@163.com (P.Y.); gwl201027@163.com (W.G.); hinamukhtar90@hotmail.com (H.M.); 18973183768@163.com (S.L.); zhengyanwen126@126.com (Y.Z.); zhongyiling321@163.com (Y.Z.); 13345066328@163.com (H.C.); 13969597234@163.com (J.L.)

**Keywords:** alginate oligosaccharides, enzymolysis, gut health

## Abstract

High viscosity of alginate means a relatively low substrate concentration, which limits the efficiency of hydrolysis, resulting in one of the main challenges for the large-scale production of alginate oligosaccharides (AOS). In this study, a pilot-scale degradation product (PSDP) of the recombinant enzyme 102C300C-Vgb was produced for the first time at a substrate concentration of up to 20% sodium alginate. The optimal conditions for SA digestion were: enzyme dosage of 25 U/g, enzymatic temperature of 45 °C, enzymatic pH of 7.0, and enzymatic time of 24 h. Under these conditions, the yield of enzymatic hydrolysis was consistently in the range of 69% to 70%. The average molecular weight (Mw) of PSDP was 1496.36 Da, mainly containing oligosaccharides with degrees of polymerization ranging from 2 to 4. The low-Mw PSDP was subsequently applied in the diet of sea cucumber *Stichopus japonicus*. The results showed that the body wall weight of *S. japonicus* increased significantly after 40 days of feeding with a 0.09% PSDP-supplemented diet. Furthermore, PSDP-supplemented diet significantly increased the thickness of the serosal and submucosal layers and the width folds of mucosa of the sea cucumber gut. The abundance of pathogenic bacteria was reduced effectively, and that of beneficial bacteria increased significantly after being fed with PSDP. The results demonstrated that PSDP can serve as a digestive health enhancer for sea cucumbers, promoting their healthy growth.

## 1. Introduction

Sea cucumbers *Stichopus japonicus* (also called *Apostichopus japonicus*) are a prevalent group of marine invertebrates [1,2]. This species is also extensively cultivated in China, with production volumes reaching 248,000 tons in 2022, underscoring its significant economic value [3]. Sea cucumbers are rich in bioactive compounds, including polysaccharides and collagen, demonstrating a range of biological activities such as anticancer, antihypertensive, antibacterial, and antioxidant effects [4]. In particular, sea cucumber collagen is free from health-related risks and religious restrictions, making it a promising alternative to mammalian collagen [5].

However, intensification of aquaculture systems, unfavorable environmental conditions, and poor management practices have significantly exacerbated disease outbreaks in aquaculture farms, thus constraining the development of the sea cucumber farming industry [6]. In routine management, antibiotics are commonly used to control bacterial diseases, but their overuse can lead to the emergence of antibiotic-resistant pathogenic strains, posing a threat to the sustainability of sea cucumber farming [7,8]. Consequently, there is an urgent need to identify efficient, safe, and sustainable alternatives to antibiotics to promote the growth and yield of *S. japonicus*. Research indicates that strategic addition of certain prebiotics or bioactive compounds can enhance feed quality, optimize the gut microecological environment, and promote organism growth and health [9,10]. Consequently, external interventions, such as the incorporation of prebiotics to maintain the balance of the microbial flora in the gut, play a crucial role in the cultivation of *S. japonicus* [11].

The prebiotics extracted from brown algae have demonstrated promising activity in the aquaculture industry [12]. Alginate oligosaccharides (AOS), the low molecular weight degradation products of alginate from brown algae and bacteria, are characterized by their improved solubility, high safety profile, and remarkable stability [13,14]. Recent studies have demonstrated that dietary supplementation with AOS also reduces *Salmonella* colonization, improving gut barrier function and poultry production performance [15,16]. A moderate amount of AOS can improve lipid metabolism and immune responses in aquatic animals, alter gut bacterial communities, and aid in combating bacterial infections [17]. Furthermore, AOS, as a dietary supplement, can enhance growth performance, non-specific immune response, and disease resistance of aquatic animals [18]. Collectively, these findings underscore the potential of AOS as a feed additive with substantial promise in improving the health and disease resistance of farmed animals. Currently, enzyme hydrolysis, a key technique in the biological method, is considered an effective strategy for the depolymerization of polysaccharides to produce specific oligosaccharides with defined degrees of polymerization [19], offering advantages such as mild reaction conditions, avoidance of radiation, low energy consumption, and high yield compared to chemical and physical methods [20,21,22]. However, the high viscosity of alginate often limits substrate concentration during enzymatic hydrolysis, resulting in reduced AOS yields [23].

In this study, the degradation process of sodium alginate was optimized using a highly active alginate lyase 102C300C-Vgb and scaled up for the pilot-scale experiment to improve substrate concentration during enzyme hydrolysis and result in high product yield. Furthermore, the pilot-scale degradation product (PSDP) was conducted with a detailed analysis of the structure and composition, and its effectiveness was evaluated in promoting the healthy aquaculture of *S. japonicus*.

## 2. Results and Discussion

### 2.1. Optimal Enzymatic Hydrolysis Conditions

The enzymatic preparation of AOS is considered an efficient, energy-saving, and environmentally friendly method. Furthermore, alginate lyase activity is critical to the efficiency of AOS production [24]. Additionally, increasing substrate concentration can enhance the AOS yield within a single batch of enzymatic hydrolysis, significantly reducing industrial production costs [25]. However, the high substrate concentration severely increases the viscosity of the SA solution, limiting the mobility of alginate lyase and affecting its ability to interact with the substrate [23]. Furthermore, current alginate lyases suffer from poor substrate specificity, low activity, and limited stability, failing to meet the demands of industrial production [26].

**Figure 1:**
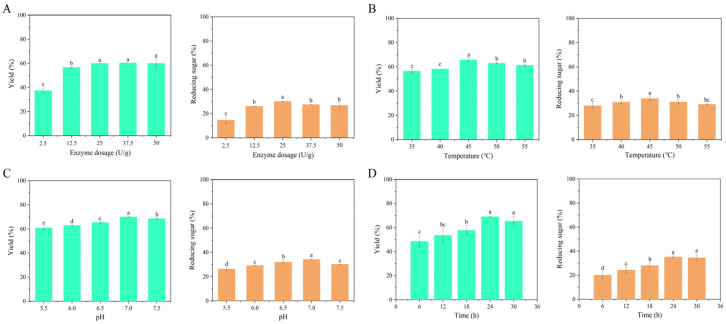
The yield and proportion of reducing sugar of digested products under different conditions. (**A**) Enzyme dosage; (**B**) Temperature; (**C**) pH; (**D**) Time. Different letters displayed above the bars on the column chart denote significant differences among treatments (*n* = 3) (mean ± SE).

In this study, the substrate concentration of 20% SA was hydrolyzed by high-activity enzyme 102C300C-Vgb with five different dosages. As shown in Figure 1A, the yield of enzymatic hydrolysis increased with the addition of enzyme dosage. When the enzyme dosage reached 25 U/g, the yield was 59.98 ± 0.03%. Further, an increase in enzyme addition did not result in a significant change in yield. Similarly, the results for the proportion of RSC (Reducing sugar content) indicated that when the enzyme dosage exceeded 25 U/g, the RSC obtained from enzymatic hydrolysis decreased (Figure 1A), demonstrating that an excessive dosage of enzyme failed to further enhance enzymatic hydrolysis efficiency. Therefore, 25 U/g was chosen as the optimal enzyme dosage.

The selection of temperature in the enzymatic hydrolysis process of alginate lyase was extremely important. To determine the optimal hydrolysis temperature, this study set up five temperature gradients. As shown in Figure 1B, the yield of enzymatic hydrolysis increased with temperature. When the temperature reached 45 °C, the yield was 65.72 ± 1.62%. Further increasing the temperature resulted in a significant decrease in yield (*p* < 0.05). In the same way, the results for the RSC indicated that when the hydrolysis temperature exceeded 45 °C, the RSC obtained from enzymatic hydrolysis decreased (Figure 1B), demonstrating that the enzyme became denatured and lost activity at excessive temperature.

Most known alginate lyases exhibited optimal catalytic activity at pH between 6.0 and 8.0 [27]. To determine the optimal hydrolysis pH, this study set up five pH gradients. The highest yield of enzymatic hydrolysis (70.01 ± 0.33%) was observed at pH 7.0 (Figure 1C). The results also indicated that the RSC obtained from enzymatic hydrolysis at pH 7.0 was significantly higher than at other pH conditions (*p* < 0.05) (Figure 1C), demonstrating that this alginate lyase preferred a neutral environment, and that both excessively high and low pH negatively affect its enzymatic efficiency.

To determine the optimal hydrolysis time, this study set up five hydrolysis time gradients. As shown in Figure 1D, the yield of enzymatic hydrolysis increased with time. When the hydrolysis time reached 24 h, the yield was highest, at 69.07 ± 0.06%, which was consistent with the results for the RSC (Figure 1D). Most known alginate lyases had poor thermal stability, exhibiting optimal catalytic activity only between 30 °C and 40 °C. Currently, there are few thermophilic alginate lyases, with recombinant alginate lyase produced by thermophilic degrading bacteria showing an optimal catalytic temperature of 50 °C. However, after reacting at 50 °C for 30 min, it lost 58% of its initial activity [28]. The alginate lyase 102C300C-Vgb used in this study can maintain its activity for 24 h at 45 °C, indicating its thermal stability [27].

In summary, optimal conditions for SA digestion using the recombinant enzyme 102C300C-Vgb with a substrate concentration of 20% SA were: enzyme dosage of 25 U/g, enzymatic temperature of 45 °C, enzymatic pH of 7.0, and enzymatic time of 24 h. Under these conditions, the yield of enzymatic hydrolysis was consistently in the range of 69% to 70%.

### 2.2. Structural Analysis of Degradation Product

#### 2.2.1. FTIR Analysis

**Figure 2:**
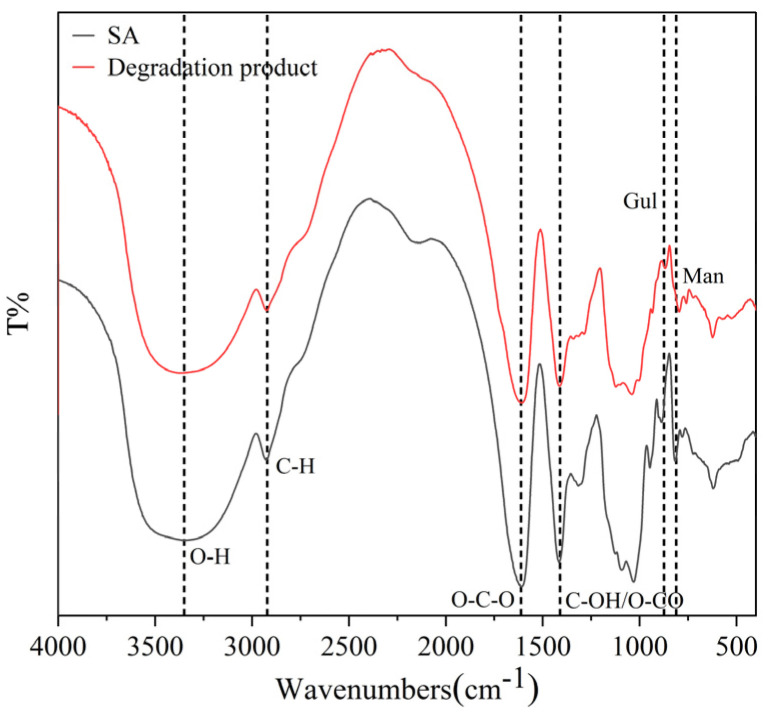
FTIR analyses of SA and its degradation product.

The degradation product was compared with SA using FTIR. In the IR spectrum (Figure 2), the peak at 3369.53 cm^−1^ corresponded to O-H stretching vibrations, while the peak at 2924.23 cm^−1^ was attributed to C-H stretching vibrations. The peaks at 1608.59 cm^−1^ and 1414.86 cm^−1^ represented asymmetrical and symmetrical O-C-O stretching vibrations, respectively. Additionally, the peaks at 868.95 cm^−1^ and 796.66 cm^−1^ were indicative of the presence of mannuronic acid and guluronic acid, respectively, according to previous findings [29]. The results indicate that the product retained the characteristic features of alginate, with no significant alterations observed in the spectra of the side groups [27].

#### 2.2.2. HPLC Analysis of Degradation Product

**Figure 3:**
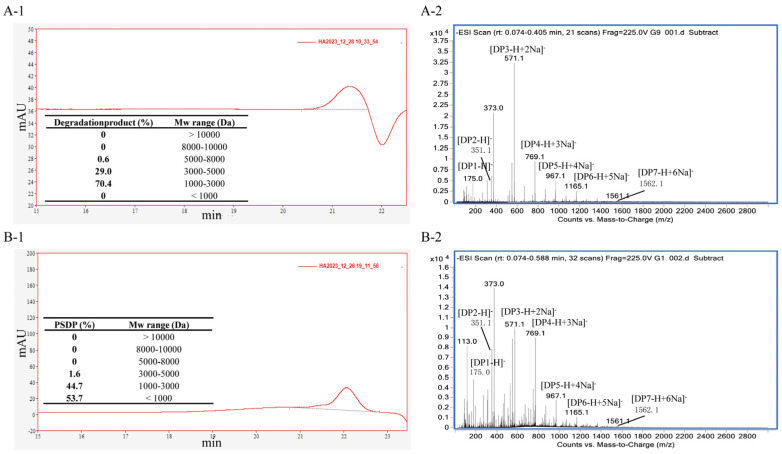
The Mw distribution (**1**) and ESI-MS (**2**) of the degradation product (**A**) and PSDP (**B**).

The Mw distribution of the degradation product was shown in Figure 3(A-1). Based on the elution time and the standard curve, the Mw range of the product was 1934–5418 Da, with an average Mw of 2575 Da. Among these, the fraction with Mw between 1000–3000 Da represented the largest proportion at 70.40%, followed by the fraction with Mw between 3000–5000 Da, which accounted for 29%.

#### 2.2.3. ESI-MS Analysis of Degradation Product

The ESI-MS spectrum for the degradation product is shown in Figure 3(A-2), indicating that the product was mainly composed of oligosaccharides with degrees of polymerization ranging from 2 to 4. AOS with this degree of polymerization was considered to possess significant biological activity [30]. The ions at 351.1 *m*/*z*, 571.1 *m*/*z*, 769.1 *m*/*z*, 967.1 *m*/*z*, and 1165.1 *m*/*z* represented unsaturated disaccharide (374 Da), unsaturated trisaccharide (572 Da), unsaturated tetrasaccharide (770 Da), unsaturated pentasaccharide (968 Da), and hexasaccharide (1166 Da), respectively. It verified that 102C300C-Vgb cleaved the glycosidic bonds in SA by β-elimination reaction.

### 2.3. Preparation and Compositional Analysis of PSDP

Currently, the high viscosity of the SA substrate imposes a significant limitation on the substrate concentration during the enzymatic production of AOS (typically below 5% *w*/*v* SA). Conventional approaches, such as increasing temperature, have proven inadequate in addressing this issue [31,32]. In this study, the PSDP was demonstrated at a substrate concentration of 20% *w*/*v* SA with a yield of 70%. Despite certain losses during production (e.g., residuals in pipelines and losses in the spray tower), this yield was generally consistent with the results obtained from high-concentration enzymatic hydrolysis of SA in laboratory optimization experiments, presenting substantial commercial potential.

Sensory, physicochemical, and contaminant analyses were performed on the PSDP, with results presented in Table 1. The color of the product changed from pale yellow-white to pale yellow-brown, attributed to the formation of unsaturated double bonds during the enzymatic hydrolysis process, which darkened the product [23]. The AOS content and water content in the product were 66.7 ± 0.1% and 5.10 ± 0.85%, which was influenced by factors such as the solid content of the solution before spray drying, the inlet and outlet temperatures of the spray tower. These parameters could affect the storage stability of the product [33]. The ash content of PSDP was 20.95 ± 3.32%, with a total arsenic content of 0.29 ± 0.01 mg/kg. Furthermore, no lead or *Salmonella* was detected, all of which met the standards for the use of feed additives.

### 2.4. Structural Analysis of PSDP

#### 2.4.1. HPLC Analysis of PSDP

Based on the elution time and the standard curve, the Mw range of PSDP was 830.51–4076.73 Da, with an average Mw of 1496.36 Da (Figure 3(B-1)). Among these, molecules with an Mw below 1000 Da constituted the largest proportion, accounting for 53.70%, followed by those in the 1000–3000 Da range, comprising 44.70%. The average Mw of PSDP was lower than that of the degradation product prepared in the laboratory, with a higher proportion of low-Mw fragments.

#### 2.4.2. ESI-MS Analysis of PSDP

The structural diagram of the PSDP was shown in Figure 3(B-2). Combined with the analysis result (Figure 3(A-1,A-2)), this further confirmed that PSDP underwent more extensive enzymatic hydrolysis compared to the degradation product prepared under laboratory conditions. The results might be attributed to more clearly defined reaction conditions in pilot-scale equipment under the same process parameters, such as the stirring speed in the reaction vessel and heating efficiency in the enzymatic hydrolysis tank.

### 2.5. Evaluation of the Feeding Effect of PSDP

#### 2.5.1. Growth Performance

**Figure 4:**
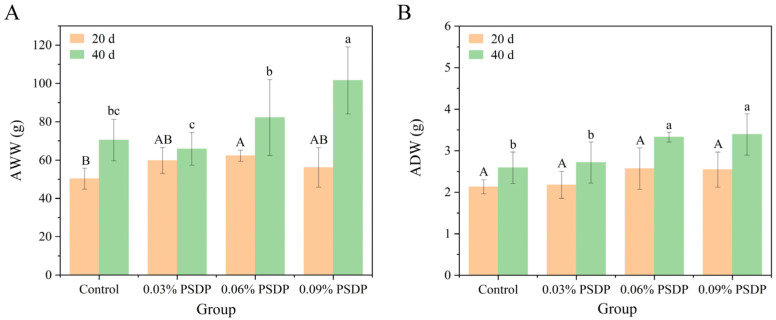
The changes of average wet weight (**A**) and average dry weight in body wall (**B**) of *S. japonicus* in different feeding groups. Different letters (uppercase letters: 20 days of cultivation; lowercase letters: 40 days of cultivation) displayed above the bars on the column chart denote significant differences among treatments (*n* = 10) (mean ± SE).

AOS exhibits excellent solubility, strong stability, ease of absorption, safety, and nontoxicity, along with a range of biological activities. These properties have led to their widespread application in fields such as biotechnology, medicine, food, and agriculture [19,34,35,36]. In the field of animal husbandry, research on AOS has primarily focused on their biological functions in poultry, piglets, and aquatic animals [15,37,38]. In this study, three proportions of PSDP were added to commercial feed to assess its growth-promoting effects on the sea cucumbers *S. japonicus*. Changes in the AWW of sea cucumbers after 20 days and 40 days of cultivation are shown in Figure 4A. After 20 days of cultivation, the wet weight of sea cucumbers fed with different concentrations of PSDP increased compared to the control group (50.29 ± 5.48 g), with the 0.06% PSDP group showing the highest weight of 62.26 ± 2.89 g (*p* < 0.05). After 40 days, the AWW of the individuals in the 0.09% PSDP group reached 101.57 ± 17.46 g, significantly higher than that of the other groups (*p* < 0.05) and 44.30% higher than that of the control group. The 0.06% PSDP group followed, with a wet weight of 82.19 ± 19.75 g. As the primary defense mechanism against pathogens, the body wall of the sea cucumber exhibits antibacterial properties and demonstrates a significant inhibitory effect on both bacteria and fungi [39]. The ADW results of the body walls for each group were presented in Figure 4B. After 20 days of cultivation, the dry weight of body walls in the groups fed by PSDP showed varying degrees of increase compared to the control group. The highest was observed in the 0.06% PSDP group (2.57 ± 0.50 g), followed by the 0.09% PSDP group (2.54 ± 0.42 g). After 40 days of cultivation, the 0.06% PSDP group exhibited a dry weight of body wall of 3.33 ± 0.12 g, an increase of 28.47% compared to the control group (2.59 ± 0.38 g) (*p* < 0.05), while the 0.09% PSDP group showed a dry weight of 3.39 ± 0.50 g, marking a 30.91% increase (*p* < 0.05). Combined with the AWW results (Figure 4A), these findings demonstrated that PSDP, as a feed additive, effectively promoted the growth of sea cucumbers *S. japonicus*. Gao [40] found that the addition of 4 g/kg of AOS enriched with polyM fragments improved the growth performance of *A. japonicus*, while higher doses had a negative impact on growth. In our experiment, the highest PSDP concentration used was 0.09%, which did not reach the levels reported to be excessive.

#### 2.5.2. Body Wall Composition

##### Protein Aspect

**Figure 5:**
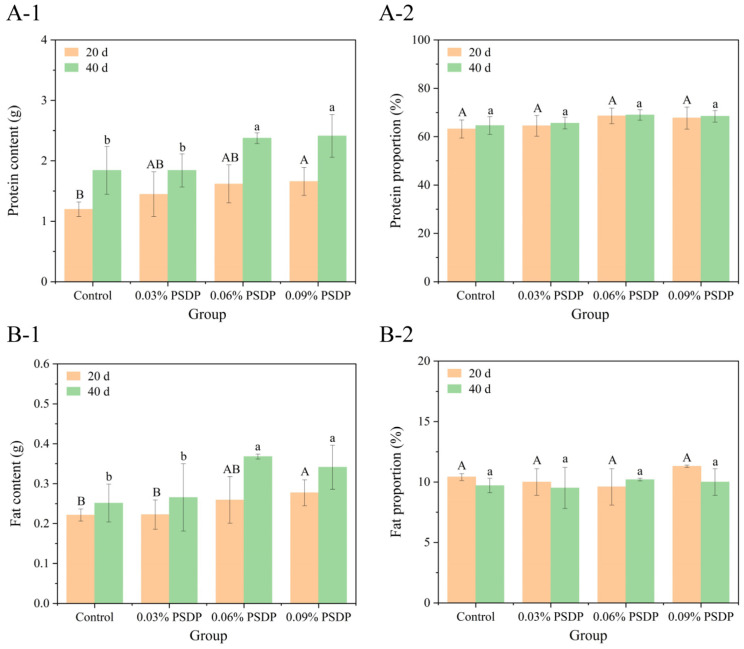
The changes of the protein (**A**)/fat (**B**) content (**1**) and proportion (**2**) in body wall of *S. japonicus* in different feeding groups. The protein/fat proportion of the body wall was calculated by dividing the protein/fat content of the individual by the dry weight of the body wall. Different letters (uppercase letters: 20 days of cultivation; lowercase letters: 40 days of cultivation) displayed above the bars on the column chart denote significant differences among treatments (*n* = 10) (mean ± SE).

The chemical composition of sea cucumbers can vary significantly across species, dietary sources, aquaculture practices, and geographic regions [41,42,43]. Changes in the protein content of the body walls of sea cucumbers during cultivation were shown in Figure 5(A-1). After 20 days of cultivation, all experimental groups fed with PSDP exhibited varying degrees of increase in total protein content of body walls. In particular, the total protein content in the body walls of sea cucumbers fed with 0.09% PSDP reached 1.66 ± 0.23 g, significantly higher than that of the control group at 1.20 ± 0.12 g (*p* < 0.05). After 40 days of cultivation, the protein content in the body walls of sea cucumbers fed with the standard diet increased to 1.84 ± 0.39 g, while the 0.06% and 0.09% PSDP groups saw increases to 2.38 ± 0.09 g and 2.41 ± 0.35 g, respectively, representing 1.29 and 1.31 times that of the standard diet group. Taking into account the ADW results, the proportion of proteins in the body wall of the individual sea cucumber was calculated (Figure 5(A-2)). Throughout the cultivation period, the protein proportion remained between 60% and 70%, with no significant differences among groups. This suggested that PSDP feeding did not affect the proportion of protein in the body walls of sea cucumbers. Instead, protein content increased with the growth in body weight, indirectly confirming the growth-promoting effect of PSDP on sea cucumbers. Furthermore, previous research has demonstrated that the protein content in the body wall of commercial sea cucumber species typically exceeds 40%, positioning them as a superior source of crude protein relative to most of the seafoods currently utilized [44]. In this study, cultured *S. japonicus* fed with PSDP exhibited protein levels greater than 60% of dry weight, exceeding other species of sea cucumbers such as *Thelenota anax*, *Holothuria spinifera*, *Holothuria scabra*, and *Bohadschia* sp. 1 [43,45,46].

##### Fat Aspect

Changes in the fat content of the body walls of sea cucumbers during cultivation were shown in Figure 5(B-1). After 20 days of cultivation, the group fed with 0.09% PSDP exhibited the highest average fat content in the body walls at 0.28 ± 0.033 g, which was significantly higher than that of the control group (0.22 ± 0.015 g) (*p* < 0.05). After 40 days of cultivation, the fat content in sea cucumbers fed with 0.06% and 0.09% PSDP increased to 0.37 ± 0.01 g and 0.34 ± 0.06 g, respectively, both significantly higher than that of the control group (*p* < 0.05). Based on the ADW results, the proportion of fat in the body wall of individual sea cucumbers was calculated (Figure 5(B-2)). The fat content of sea cucumbers typically ranges between 0.3% and 1.9% [43]. Throughout the cultivation period, the fat proportion remained around 10%, with no significant differences among groups. This is directly related to the species of sea cucumbers [47].

##### Amino-Acid Composition

Table 2 illustrates the changes in the amino acid composition of the body walls of sea cucumbers as a percentage of the total weight during cultivation. Glutamic acid and glycine were the most abundant amino acids, followed by aspartic acid, alanine, and arginine, while cysteine and histidine were the least abundant, at levels similar to Li’s report [47]. However, analyses of *A. japonicus* from Korea revealed that the predominant amino acids in its body wall were glutamic acid, arginine, aspartic acid, and glycine. These variations may be attributed to differences in habitat and feeding conditions [48]. The most abundant amino acids (glutamic acid and glycine) in the samples play a significant role in immune regulation [44], making all cultured sea cucumbers have a greater market value. Furthermore, the low Lys/Arg ratio of a protein in the samples contributes to the hypocholesterolemic effect [49], making it an ideal dietary option for individuals with high cholesterol levels. Comparing the results across the four groups on 20 days and 40 days, no significant increases or decreases in amino acid composition percentages were observed, suggesting that the amino acid composition of the body walls of sea cucumbers was not affected by PSDP supplementation. This was consistent with the results for the proportions of protein and fat.

#### 2.5.3. Gut Health

##### Intestinal Morphology Analysis

**Figure 6:**
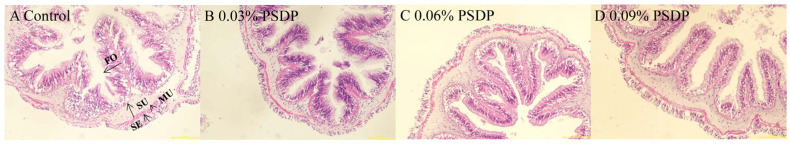
Intestinal tissue sections of *S. japonicus* fed diets with different PSDP levels. SE: Serosal layer; MU: Muscularis propria; SU: Submucosa; FO: Fold width.

Intestinal morphology is a crucial indicator of intestinal health [50], closely related to the ability to digest and absorb nutrients [51]. Incorporation of AOS into feed has been consistently shown to promote intestinal development in farmed animals [52,53]. Compared to the control group, the sea cucumbers fed with PSDP exhibited a more regular and intact intestinal structure (Figure 6). As shown in Table 3, the thickness of the serosa layer and the width of the mucosal fold in the 0.09% PSDP group were significantly greater than those in the other groups (*p* < 0.05). Additionally, the thickness of the submucosa in all PSDP-supplemented diet groups was markedly higher than that in the control group (*p* < 0.05). However, there was no significant difference in the thickness of the muscularis propria among the groups. In this study, PSDP supplementation increased the thickness of the intestinal wall and the width of the mucosal folds in sea cucumbers, indicating enhanced intestinal activity and increased feed intake. The increase in intestinal folds of sea cucumbers is likely to enhance carbohydrate absorption and utilization, enabling the conversion of a portion of the increased feed intake into glycogen, thus maintaining carbohydrate balance [54].

##### Gut Microbial Diversity Analysis

Bacterial communities constitute a significant portion of the gut microbiota in sea cucumbers [55], playing a crucial role in digestion and nutrient absorption, as well as providing more than 70% of the energy requirement of sea cucumbers [56]. Additionally, the gut microbiota is closely related to the overall health and growth of sea cucumbers [57]. Based on the previous findings, the control group (CT), the 0.06% PSDP group (PSDP6), and the 0.09% PSDP group (PSDP9) were selected to assess the impact of PSDP on the intestinal microbiota of sea cucumbers.

The number of ASVs, as well as the Shannon, ACE, Chao1, and Simpson indices obtained for each sample from each group, is presented in Table S1. Studies have shown that the addition of AOS enriched with polyM fragments to sea cucumber diets could balance the abundance and diversity of the gut microbiota [40]. Compared to the CT group, there were no significant differences in various diversity indices in the PSDP-treated groups, indicating that the PSDP6 and PSDP9 did not affect the diversity of the gut microbiota in sea cucumbers.

A total of 32 bacterial phyla, 106 classes, 263 orders, 428 families, 901 genera, and 1385 species were identified in all samples. The Venn diagram showed that 360 ASVs were shared between the CT and the PSDP-treated groups (Figure S2).

At the phylum level, the predominant bacterial phyla detected in the gut of sea cucumbers were Firmicutes (45.5 ± 4.27%), followed by Bacteroidetes (22.98 ± 7.95%), Proteobacteria (12.49 ± 3.07%), and Actinobacteria (11.08 ± 0.74%) (Table S2). Upon administration of PSDP6 and PSDP9, the relative abundance of Bacteroidetes in the gut exhibited an upward trend in all experimental groups. Previous studies have demonstrated that Bacteroidetes are capable of degrading complex organic materials, such as polysaccharides and hydrocarbons [58]. Additionally, AOS has been found to upregulate Bacteroidetes, which can play a crucial role in the probiotic activity of AOS in vivo [59]. Consistent with these findings, this study revealed that the relative abundance of Bacteroidetes increased to varying extents in the gut of sea cucumbers fed with PSDP, indicating that PSDP could positively contribute to gut health in sea cucumbers. In particular, the relative abundance of Firmicutes in the PSDP9 group increased compared to the CT group. In contrast, the relative abundances in Proteobacteria, Actinobacteria, and Fusobacteria were reduced (Figure S3). Firmicutes are involved in the breakdown of carbohydrates and the degradation of the components of the cell wall, leading to the production of short-chain fatty acids that serve as vital nutrients for the host [60]. Furthermore, certain Firmicutes have been shown to enhance host immunity by upregulating the expression of immune-related genes [61].

**Figure 7:**
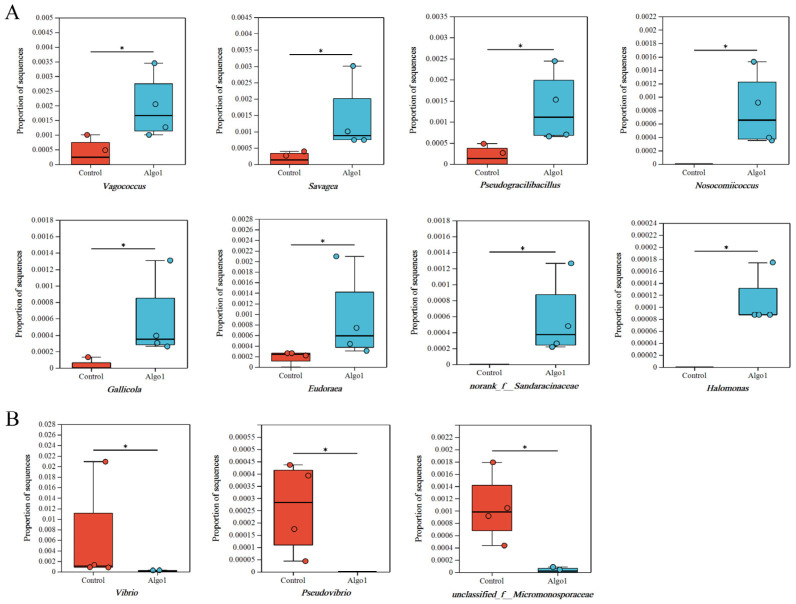
Analysis of differences in abundance between PSDP6 and the CT at the genus level: (**A**). compared to CT, the bacteriuml communities of increased abundance in PSDP6; (**B**). compared to CT, the bacteriuml communities of decreased abundance in PSDP6. Values with * were *p* < 0.05 between two treatments (*n* = 4) (mean ± SE).

**Figure 8:**
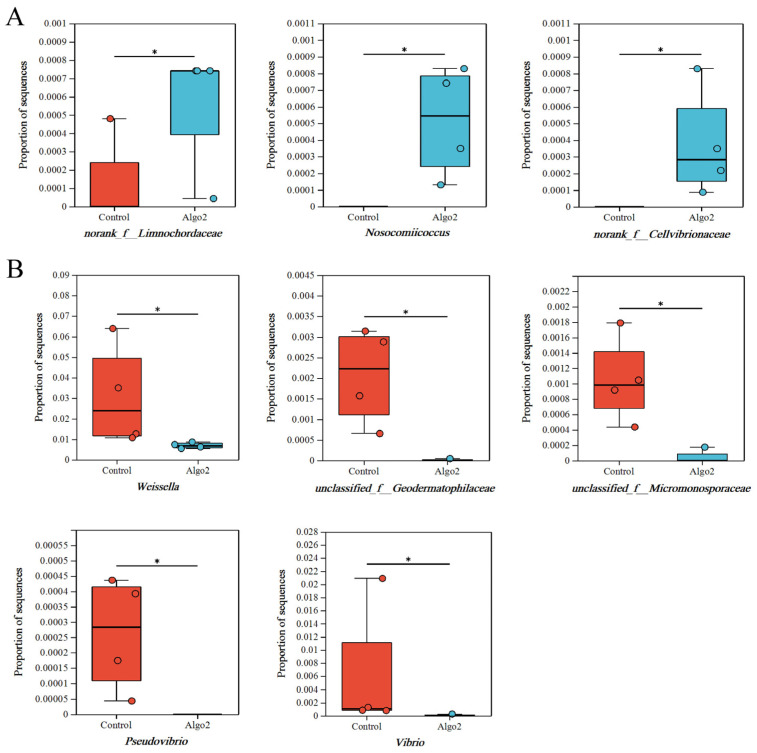
Analysis of differences in abundance between PSDP9 and the CT at the genus level: (**A**). compared to CT, the bacteriuml communities of increased abundance in PSDP9; (**B**). compared to CT, the bacteriuml communities of decreased abundance in PSDP9. Values with * were *p* < 0.05 between two treatments (*n* = 4) (mean ± SE).

At the genus level, significant differences were observed in the relative abundance of microbial communities in the gut of sea cucumbers fed with PSDP compared to the CT group. In the PSDP6 group, the relative abundances of the genera *Vagococcus*, *Savagea*, *Pseudogracilibacillus*, *Nosocomiicoccus*, and *Gallicola* in Firmicutes, as well as the genus *Eudoraea* in Bacteroidetes, and the genera *norank f Sandaracinaceae* and *Halomonas* in Proteobacteria, increased significantly (*p* < 0.05). Conversely, the relative abundances of the genera *Vibrio* and *Pseudovibrio* in Proteobacteria, and *unclassified f Micromonosporaceae* in Actinobacteria, decreased significantly (*p* < 0.05) (Figure 7). In the PSDP9, the relative abundances of genera *norank f Limnochordaceae* and *Nosocomiicoccus* in Firmicutes, as well as the genus *norank f Cellvibrionaceae* in Proteobacteria, elevated significantly (*p* < 0.05). In contrast, the relative abundances of the genus *Weissella* in Firmicutes and genera *unclassified f Geodermatophilaceae*, *unclassified f Micromonosporaceae*, *Pseudovibrio*, and *Vibrio* in Proteobacteria reduced significantly (*p* < 0.05) (Figure 8). Gut microbiota plays a crucial role in maintaining gut health by facilitating digestion and regulating the homeostasis of the organism’s micro-ecological system [62,63]. They can reshape the beneficial effects of AOS on aquatic animals [64]. As shown in Figure 7 and Figure 8, feeding sea cucumbers with PSDP6 and PSDP9 consistently promoted the growth of the genus *Nosocomiicoccus*, while simultaneously inhibiting the relative abundance of the genera *Vibrio*, *Pseudovibrio*, and *unclassified f Micromonosporaceae*. *Vibrio* is generally regarded as a primary opportunistic pathogen that contributes to disease and mortality in aquatic animals [65]. Moreover, acting as the primary pathogenic bacteria for sea cucumbers, the significant reduction in the abundance of *Vibrio* in the samples mitigates the risk of disease in the cultivation of *S. japonicus* [66]. *Pseudovibrio* strains can synthesize secondary metabolites and contribute essential cofactors to their hosts through nonribosomal peptide synthetase and polyketide synthase pathways [67]. The observed reduction in the abundance of *Pseudovibrio* in the samples can be attributed to factors such as the farmed species and environmental conditions [68]. There were numerous reports on the interaction between AOS and gut microbiota, as well as its regulatory effects on various diseases. AOS exhibited functions in microbiota modulation similar to those of alginate. For instance, low Mw alginate extracted from kelp not only reduced obesity symptoms in HFD-fed BALB/c mice but also modulated the gut microbiota by increasing the abundance of Bacteroidales and decreasing *Clostridia*, thus improving the physiological state of the mice [64]. The regulation of the gut microbiota also contributed to the anti-obesity effects of AOS, significantly promoting the growth of *Akkermansia muciniphila*, *Lactobacillus reuteri*, and *Lactobacillus gasseri*. Furthermore, the AOS intervention significantly increased the concentration of short-chain fatty acids (SCFAs), such as acetate, propionate, and butyrate, while reducing endotoxin levels [69]. In colitis mouse models, treatment with unsaturated AOS inhibited immune damage and maintained mucosal barrier function by modulating gut microbiota, increasing the abundance of Firmicutes and Actinobacteria, while decreasing the abundance of Bacteroidetes [18]. Additionally, AOS supported the growth of beneficial gut bacteria [70,71]. Collectively, the results suggested that AOS promoted the growth of beneficial bacteria and enhanced gut health in the gut of *S. japonicus*. This could potentially bolster growth and augment non-specific immunity in *S. japonicus*.

## 3. Materials and Methods

### 3.1. Experimental Materials

The alginate lyase used in this study was 102C300C-Vgb (203 U/mL), developed by the Laboratory of Marine Microbial Engineering at Ocean University of China [72]. Sea cucumbers *S. japonicus* and experimental diets were provided by Yantai Huakang Biomedical Technology Co., Ltd., Yantai, Shandong (China).

### 3.2. Optimization of Enzymatic Conditions

#### 3.2.1. Enzyme Dosage

Different enzyme dosages (each g of raw material: 2.5 U, 12.5 U, 25 U, 37.5 U, and 50 U) of 102C300C-Vgb were mixed with 20% sodium alginate (SA) and incubated at 45 °C, pH 7.0, for 24 h in a 250 mL shaker to obtain the hydrolysis sample. Subsequently, the sample yield and reducing sugar content were determined to investigate the effect of the amount of addition.

#### 3.2.2. Enzymatic Temperature

The enzymatic temperature (35 °C, 40 °C, 45 °C, 50 °C, and 55 °C) was optimized based on the optimal amount of enzyme addition (25 U/g). Other operations were the same as those in the above section.

#### 3.2.3. Enzymatic pH

Based on the above optimum enzymatic conditions (enzyme dosage of 25 U/g and enzymatic temperature of 45 °C), the pH of the enzymatic hydrolysis system was adjusted to 5.5, 6.0, 6.5, 7.0, and 7.5, respectively, by varying the amount of phosphate added to the culture medium.

#### 3.2.4. Enzymatic Time

The enzymatic time (6 h, 12 h, 18 h, 24 h, and 30 h) was optimized according to the optimum enzymatic conditions mentioned above (enzyme dosage of 25 U/g, enzymatic temperature of 45 °C, and enzymatic pH of 7.0). The sample was subsequently vacuum freeze-dried for further analysis.

### 3.3. 250-L Enlargement Experiment

**Figure 9:**
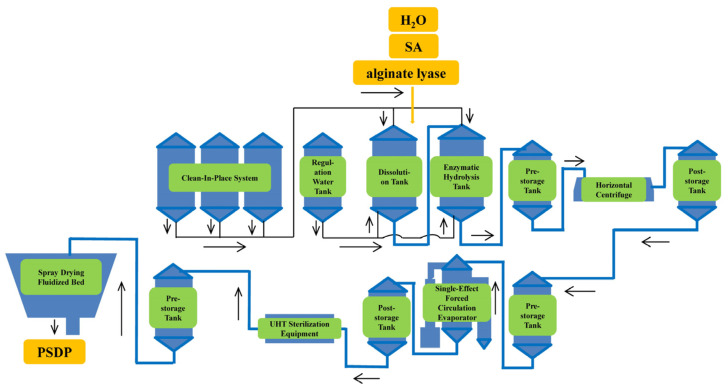
Flow chart of pilot production equipment.

The PSDP preparation process optimized at the laboratory level was scaled up for pilot production using the equipment shown in Figure 9. In this process, 50 kg of SA was mixed with 200 L of water at a material-to-water ratio of 1:4, with simultaneous addition and uniform stirring. The mixture was then subjected to enzymatic hydrolysis at 45 °C for 24 h in an enzymatic hydrolysis tank. After the hydrolysis, the temperature was raised to 85 °C for 10 min to deactivate the enzymes. The supernatant, obtained after centrifugation to remove impurities, was concentrated to one-third of its original volume, followed by UHT sterilization, and then subjected to spray drying.

### 3.4. S. japonicus Feeding Experiment

The sea cucumbers were cultured for 40 days in the sea area of Longkou Economic Development Zone (120°28′ E, 37°58′ N). Four basal diets were supplemented with a diet of 0, 300, 600, and 900 mg PSDP/kg (hereinafter referred to as the control, 0.03% PSDP, 0.06% PSDP, and 0.09% PSDP groups). Before the start of the experiment, sea cucumbers were fed the control diet for 1 week to acclimatize to the experimental conditions. Each group has two replicates, and each of similar-sized 2000 *S. japonicus* (40.0 ± 1.0 g of wet body weight) was selected out and randomly distributed into 8 aquaculture cages (length × width × height: 4.00 × 4.00 × 2.80 m), under a natural photoperiod (12 dark:12 light). The sea cucumbers were fed on experimental diets once a day at 10:00 am to ad libitum for a consecutive 40 days. The daily feeding rate was 3% of the total body weight.

### 3.5. Data Measurement

#### 3.5.1. Yield Analysis of Enzymatic Hydrolysis

The enzymatic supernatant, which has been centrifuged to remove the precipitate, was lyophilized or spray-dried. Subsequently, it was dried to constant weight using a weighing bottle. The yield of enzymatic hydrolysis was calculated according to the following formulas:

$$Yield\left(\%\right)=P*100\%/M$$

P: degradation product weight; M: SA weight.

#### 3.5.2. Reducing Sugar Content Analysis

Reducing sugar was determined by the 3,5-dinitrosalicylic acid (DNS) method [73]. 2 mL of DNS solution was added to the appropriately diluted enzymatic hydrolysate. The mixture was incubated in a boiling water bath for 2 min and ddH_2_O was added to a total volume of 15 mL. Absorbance was measured at 540 nm. Reducing sugar content (RSC) was calculated according to the following formulas:

RSC(%)=RS∗100%/M

RS: reducing sugar weight; M: SA weight.

#### 3.5.3. Fourier Transform Infrared Analysis

Fourier transform infrared (FTIR) spectra were obtained using a Magna-IR560 spectrometer (Nicolet, Madison, WI, USA). For the analysis, 2 mg of the sample was homogenized with 200 mg of KBr and subsequently compressed into a disk. Spectral data were acquired across the 4000–400 cm^−1^ range, employing a resolution of 4 cm^−1^.

#### 3.5.4. High-Performance Liquid Chromatography Analysis

The molecular weight (Mw) of the degradation product was determined using high-performance liquid chromatography on an Agilent 1260 Infinity system equipped with a PL Aquagel-OH 30 column (Agilent, Santa Clara, CA, USA). A mobile phase consisting of 200 mM NaNO_3_ and 10 mM NaH_2_PO_4_ was employed at a flow rate of 0.5 mL/min, with the column maintained at 25 °C. Detection was carried out using a refractive index detector (RID). Dextran standards with molecular weights of 1 kDa, 3.65 kDa, 5 kDa, and 12 kDa were utilized for calibration. Prior to injection, the precipitates were dissolved in the mobile phase and filtered through a 0.22 µm membrane.

#### 3.5.5. Electrospray Ionization Mass Spectrometry Analysis

The dried samples were introduced by direct infusion into the electrospray ionization source (ESI), and mass spectra (MS) were collected for elucidating the structure of the ESI-MS peaks (Agilent 1290 Infinity II-6460, Frag = 175.0 V, *m*/*z* 100–2000 amu) [27].

#### 3.5.6. Compositional Analysis of Pilot-Scale Products

Proximate composition was determined on the PSDP. The appropriate amount of the sample was placed in a clean and dry white porcelain dish for sensory evaluation. The AOS content was measured using the m-hydroxybiphenyl method [74]. Weight difference methods were used to determine moisture and ash content [75]. The particle size was calculated using an 80 mesh standard sieve pass rate. The total arsenic and lead were determined by Hydride Generation Atomic Fluorescence Spectrometry (HG-AFS) [76] and Atomic Absorption Spectroscopy (AAS) [77]. Additionally, samples were tested for the presence of *Salmonella* contamination.

#### 3.5.7. Growth Performance

Ten sea cucumbers were randomly selected from 2 cages in each group and weighed to measure the growth parameters every 20 days. The average value of each 40 individual animals was considered as a valid value for each replicate. Average wet weight (AWW) was calculated according to the following formulas:

AWW(g)=TWW/N

TWW: total wet weight; N: the number of sea cucumbers

The individuals after weighed were dissected on ice, and the body walls obtained were boiled for 5 min to prevent autolysis. The body walls were subsequently lyophilized and pulverized. The average dry weight (ADW) was calculated after taking a portion of it for constant weight:

$$ADW\left(g\right)=TDW/N$$

ADW: total dry weight; N: sea cucumber number.

#### 3.5.8. Analyses of Body Wall Compositions

The freeze-dried body walls were analyzed for protein, fat, and amino acid composition. Protein content was measured using a Kjeldahl nitrogen analyzer (SKD-100, Shanghai Peiou Analytical Instrument Co., Ltd., Shanghai, China) [78,79]. The crude fat was determined using Soxhlet extraction with petroleum ether as a solvent [80]. The amino acid composition of the samples was analyzed using a fully automated amino acid analyzer (L-8900, Hitachi High-Tech Science Corporation, Tokyo, Japan).

#### 3.5.9. Intestinal Morphometry

The mid-intestinal section (3 cm) was fixed in Bouin’s fixative solution (Phygene, Wuhan, China), embedded in paraffin blocks, sliced to 4 μm thickness sections for hematoxylin and eosin staining (H and E; Wuhan Servicebio Biotechnology Co., Ltd., Wuhan, China), and examined under a light microscope (BX41, Olympus, Tokyo, Japan). Subsequently, the images were analyzed with AJ-VERT software, version 6.0. The thickness of the intestinal serosa, the muscularis propria and submucosa, and the width of the folds were measured (six measurements of each type of structure, five sea cucumbers per tank).

#### 3.5.10. Intestinal DNA Extraction and Illumina Sequencing of 16S rRNA Genes

Total DNA was extracted from the gut using the E.Z.N.A TM bacterial Mag-Bind DNA Kit (OMEGA, Beijing, China) following the manufacturer’s instructions. DNA concentration was quantified using Qubit 3.0 (Invitrogen, Q32866, Waltham, MA, USA), while library concentration was quantified by a fluorescence instrument. Amplification and sequencing of the V3-V4 region of the 16S rRNA gene were performed using fusion primers, including 338F (5′-ACTCCTACGGGAGGCAGCAG-3′) and 806R (5′-GGACTACHVGGGTWTCTAAT-3′). PCR, PCR product purification, quantification, and high-throughput sequencing were performed using the Illumina MiSeq platform at Shanghai Majorbio Bio-Pharm Technology Co., Ltd., Shanghai, China. Bioinformatics analysis of the gut microbiota was performed using the Majorbio Cloud platform (URL accessed on 15 January 2024) (https://cloud.majorbio.com).

### 3.6. Statistical Analysis

Statistical analysis was performed using one-way analysis of variance (ANOVA) with post hoc multiple comparison LSD tests to detect significant differences.

## 4. Conclusions

In this study, recombinant alginate lyase 102C300C-Vgb was employed to achieve targeted enzymatic hydrolysis of oligosaccharides under 20% SA concentration, which is the highest substrate concentration reported to date, significantly improving the production efficiency of PSDP. In particular, when 0.06% PSDP was added to the diet of sea cucumber *S. japonicus*, the growth and gut health were all improved significantly. This study presents a novel method for the degradation of high concentrations of SA, which has good economic and application potential in industrial production and sustainable marine aquaculture.

## Figures and Tables

**Table 1:** Detection of related index of PSDP.

Index	Detection Result
Sensory requirements	
Sensation	Powder, light yellowish-brown in color, free from mold, spoilage, and off odors or smells
Physicochemical parameters	
AOS content (%)	66.7 ± 0.1
Water content (%)	5.10 ± 0.85
Crude ash content (%)	20.95 ± 3.32
Particle size (80 mesh pass rate, %)	99.50 ± 0.42
Sanitary indicators	
Total arsenic (mg/kg)	0.29 ± 0.01
Lead (mg/kg)	ND
*Salmonella* (/25g)	ND

Values expressed in mean ± SD. ND means no data.

**Table 2:** Amino-acid composition of body wall of *S. japonicus*.

20 Day	Control (%)	0.03% PSDP (%)	0.06% PSDP (%)	0.09% PSDP (%)	40 Day	Control (%)	0.03% PSDP (%)	0.06% PSDP (%)	0.09% PSDP (%)
Asp	4.60	4	4.80	4.70	Asp	4.80	4.70	4.30	4.50
Thr	2.40	2.10	2.50	2.60	Thr	2.50	2.50	2.20	2.30
Ser	2.30	2	2.40	2.50	Ser	2.30	2.30	2.1	2.30
Glu	7.20	6.20	7.40	7.06	Glu	7.40	7.30	6.60	7
Gly	6.50	5.60	6.60	7.10	Gly	6.10	6.50	5.90	6.10
Ala	3	2.60	3	3.70	Ala	2.90	3	2.70	2.70
Cys	0.60	0.40	0.50	0.80	Cys	0.40	0.30	0.30	0.30
Val	1.90	1.60	1.90	1.90	Val	2	1.80	1.60	1
Met	0.80	0.70	0.70	1.20	Met	0.90	0.80	0.70	0.40
Ile	1.40	1.50	1.50	1.90	Ile	1.50	1.30	1.20	0.70
Leu	2	2	2.20	2.70	Leu	2.40	2.20	2	2.10
Tyr	1.50	1.20	1.40	1.90	Tyr	1.40	1.30	1.20	1.90
Phe	1.50	1.20	1.50	1.50	Phe	1.50	1.30	1.30	1.80
Lys	1.90	1.50	1.90	1.80	Lys	2	1.80	1.60	1.50
His	0.50	0.50	0.60	0.70	His	0.60	0.70	0.50	0.30
Arg	3.40	2.90	3.50	3.50	Arg	3.40	2.40	3.10	2.90
Hypro	2.40	2	2.30	2.90	Hypro	2	2.40	2.10	2.20
Pro	3.20	2.80	3.30	3.20	Pro	3.10	3.40	3	2.80

The color in the table represents the change in the content of each amino acid. The redder the color, the higher the content; the whiter the color, the lower the content.

**Table 3:** Effects of dietary PSDP on intestinal morphology structure of *S. japonicus*.

Group	Serosa Layer Thickness (μm)	Muscularis Propria Thickness (μm)	Submucosa Thickness (μm)	Folds Width (μm)
Control	22.25 ± 10.37 ^b^	9.64 ± 1.7 ^b^	23.61 ± 3.42 ^b^	211.85 ± 25.8 ^c^
0.03% PSDP	25.45 ± 2.58 ^b^	9.41 ± 1.55 ^b^	37.78 ± 9.06 ^a^	184.23 ± 13.5 ^c^
0.06% PSDP	29.49 ± 9.99 ^b^	12.58 ± 2.13 ^a^	43.83 ± 7.82 ^a^	246.62 ± 23.4 ^b^
0.09% PSDP	39.72 ± 3.55 ^a^	11.92 ± 2.56 ^ab^	41.26 ± 3.89 ^a^	311.01 ± 3.99 ^a^

Different letters within a line denote significant differences (*p* < 0.05). Values expressed in mean ± SD.

## Data Availability

The original contributions presented in this study are included in the article. Further inquiries can be directed to the corresponding authors.

## Supplementary Materials

The following supporting information can be downloaded at: https://www.mdpi.com/article/10.3390/md23090339/s1, Figure S1: The principal coordinates analysis (PCoA) showing the separate distribution of gut microbiota composition on the plot among various PSDP treatment groups and the CT. Figure S2: Venn diagram showing the number of unique and shared ASVs of gut bacteria between the CT and the PSDP treatment groups. Figure S3: Relative abundance of bacteriuml communities in different samples at the top 10 phylum level. Table S1: An Illumina high-throughput sampling depth, richness, and diversity index of bacteriuml community in the gut of *S. japonicus* under different experimental groups. Table S2: Relative abundances of thirty-two different bacteria phyla under different experimental groups.

## Abbreviations

The following abbreviations are used in this manuscript:

*S. japonicus*	*Stichopus japonicus*
*A. japonicus*	*Apostichopus japonicus*
SA	Sodium alginate
AOS	Alginate oligosaccharides
PSDP	Pilot-scale degradation product
RSC	Reducing sugar content
FTIR	Fourier transform infrared
Mw	Molecular weight
AWW	Average wet weight:
ADW	Average dry weight
