# Supplementary material for: New Strategy for the Degradation of High-Concentration Sodium Alginate with Recombinant Enzyme 102C300C-Vgb and the Beneficial Effects of Its Degradation Products on the Gut Health of Stichopus japonicus
Source: Mar Drugs. 2025 Aug 25;23(9):339. doi: 10.3390/md23090339 (PMC12472054; doi:10.3390/md23090339)
Supplement: Supplementary file 1 [file marinedrugs-23-00339-s001.zip › marinedrugs-3809236-supplementary.docx]

**Supplementary Materials**

**New Strategy for the Degradation of High-Concentration Sodium Alginate and the Improvement Effects of Its Degradation Products on the Gut Health of *Stichopus japonicus***

**Ziqiang Gu ^1^, Feiyu Niu ^1^, Peng Yang ^1^, Wenling Gong ^1^, Hina Mukhtar ^1^, Siyu Li ^1^, Yanwen Zheng ^1^, Yiling Zhong ^1^, Hanyi Cui ^1^, Jichao Li ^1^, Haijin Mou** **^1,*^, Dongyu Li ^1,*^**

Affiliations:

^1^ College of Food Science and Engineering, Ocean University of China, No.1299 Sansha Road, Qingdao 266003, China

***** Correspondence: [mousun@ouc.edu.cn](mailto:mousun@ouc.edu.cn) (H.M.); [ldy1516@ouc.edu.cn](mailto:ldy1516@ouc.edu.cn) (D.L.)

**
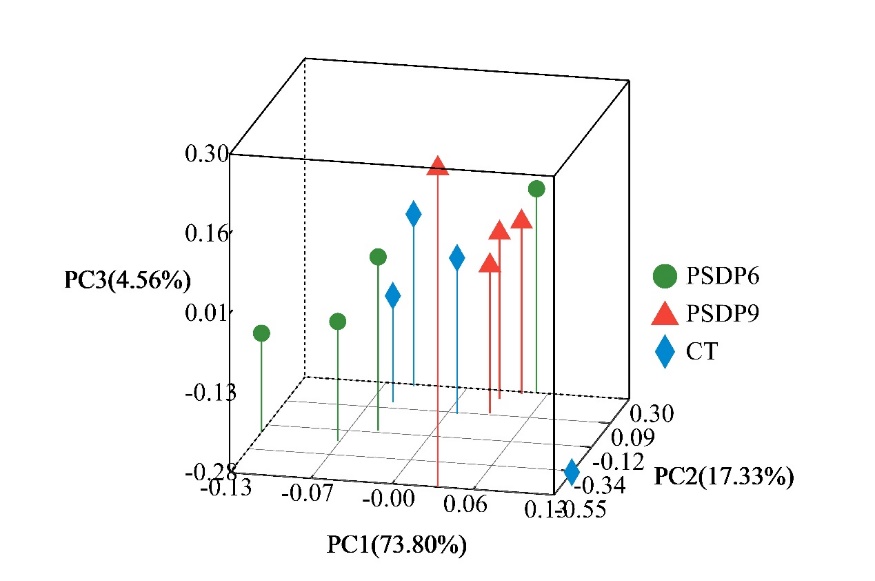
**

Figure S1 The principal co-ordinates analysis (PCoA) showing the separate distribution of gut microbiota composition on the plot among various PSDP treatment groups and the CT (N = 4).


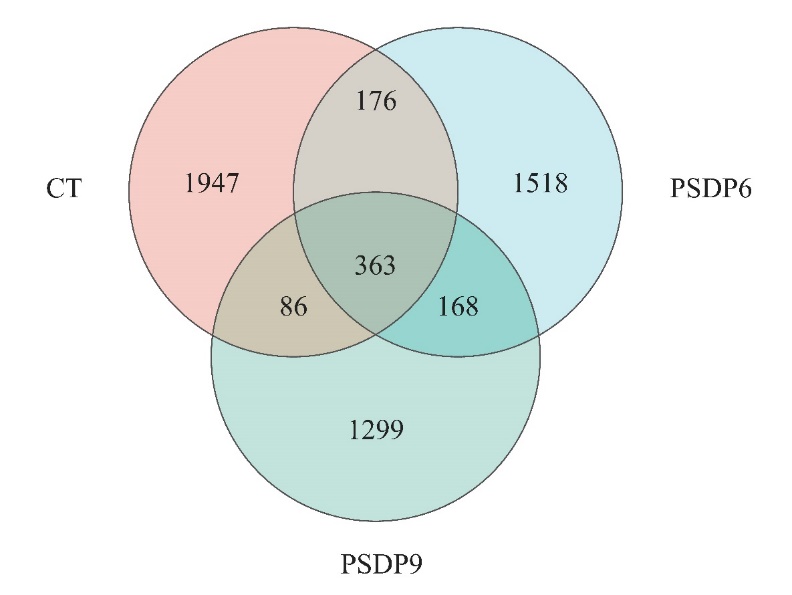


Figure S2 Venn diagram showing the number of unique and shared ASVs of gut bacteria between the CT and the PSDP treatment groups (N = 4).

**
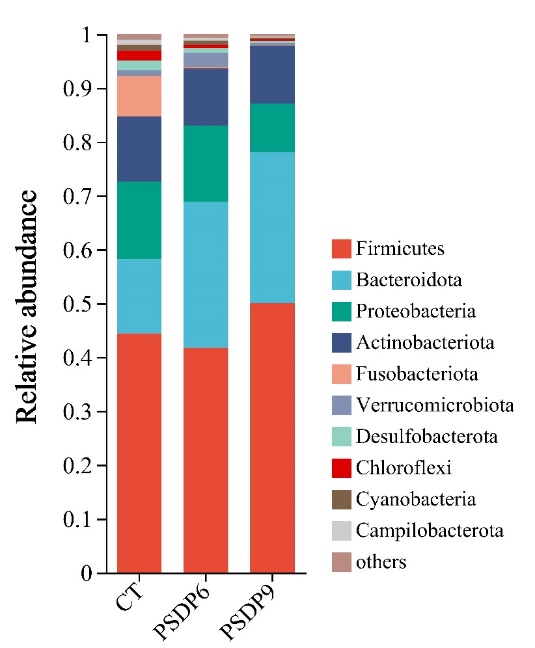
**

Figure S3 Relative abundance of bacteriuml communities in different samples at the top 10 phylum level (N = 4).

Table S1 An Illumina high-throughput sampling depth, richness and diversity index of bacteriuml community in the gut of *S. japonicus* under different experimental groups.

| Index | Control | Treat Groups of AOS | |
| --- | --- | --- | --- |
|  |  | 0.06% | 0.09% |
|  | CT | PSDP6 | PSDP9 |
| Sampling depth | | | |
| mean sequences | 63633.50 | 62974.50 | 57947.75 |
| ASVs | 2559 | 2220 | 1910 |
| goods_coverage | 100% | 100% | 100% |
| Richness index | | | |
| Sobs | 317.25 ± 96.13^a^ | 328.25 ± 38.69^a^ | 356.33 ± 22.5^a^ |
| ACE | 331.29 ± 94.09^a^ | 345.1 ± 40.55^a^ | 378 ± 25.46^a^ |
| Chao | 325.99 ± 94.23^a^ | 338.38 ± 39.91^a^ | 373.82 ± 23.39^a^ |
| Diversity index | | | |
| Shannon | 3.18 ± 0.51^a^ | 3.03 ± 0.18^a^ | 3.27 ± 0.07^a^ |

Different letters within a line denotes significant differences (*P < 0.05*). Values expressed in mean ± SD.

Table S2 Relative abundances of thirty-two different bacteria phyla under different experimental groups.

| Phylum level | Relative abundances (%) | | |
| --- | --- | --- | --- |
|  | Control | PSDP6 | PSDP9 |
| Firmicutes | 44.59 | 41.76 | 50.15 |
| Bacteroidota | 13.81 | 27.16 | 27.95 |
| Proteobacterium | 14.32 | 14.19 | 8.95 |
| Actinobacteriota | 11.93 | 10.56 | 10.76 |
| Fusobacteriota | 7.57 | 0.28 | 0.15 |
| Verrucomicrobiota | 1.10 | 2.64 | 0.38 |
| Desulfobacterota | 1.75 | 0.84 | 0.38 |
| Chloroflexi | 1.86 | 0.57 | 0.32 |
| Cyanobacterium | 1.14 | 0.78 | 0.32 |
| Campilobacterota | 0.88 | 0.52 | 0.23 |
| unclassified_k__norank_d__Bacterium | 0.28 | 0.14 | 0.090 |
| Myxococcota | 0.12 | 0.15 | 0.11 |
| Dependentiae | 0.18 | 0.076 | 0.028 |
| Acidobacteriota | 0.11 | 0.087 | 0.032 |
| Planctomycetota | 0.16 | 0.046 | 0.017 |
| Bdellovibrionota | 0.040 | 0.066 | 0.050 |
| Patescibacterium | 0.040 | 0.036 | 0.023 |
| Gemmatimonadota | 0.020 | 0.027 | 0.019 |
| Modulibacterium | 0.035 | 0.013 | 0.0022 |
| Sva0485 | 0.035 | 0.013 | 0.0022 |
| Halanaerobiaeota | 0.0055 | 0.012 | 0.0055 |
| NB1-j | 0.0055 | 0.0087 | 0.0076 |
| SAR324_cladeMarine_group_B | 0.0087 | 0.0033 | 0.0011 |
| Nitrospirota | 0.0022 | 0.0076 | 0.0044 |
| Elusimicrobiota | 0 | 0.012 | 0 |
| Deinococcota | 0.0044 | 0 | 0.0044 |
| Armatimonadota | 0.0044 | 0.0044 | 0 |
| Dadabacterium | 0 | 0.0076 | 0 |
| Methylomirabilota | 0 | 0.0033 | 0.0044 |
| Schekmanbacterium | 0.0033 | 0.0011 | 0 |
| Sumerlaeota | 0.0022 | 0.0022 | 0 |
| Entotheonellaeota | 0 | 0.0022 | 0 |
